# Developmental Letter Position Dyslexia in Turkish, a Morphologically Rich and Orthographically Transparent Language

**DOI:** 10.3389/fpsyg.2019.02401

**Published:** 2019-11-05

**Authors:** Selçuk Güven, Naama Friedmann

**Affiliations:** ^1^School of Communication Sciences and Disorders, McGill University, Montreal, QC, Canada; ^2^Department of Speech and Language Therapy, Anadolu University, Eskişehir, Turkey; ^3^Language and Brain Lab, Sagol School of Neuroscience and School of Education, Tel Aviv University, Tel Aviv, Israel

**Keywords:** developmental dyslexia, letter position dyslexia, Turkish, transparent orthography, morphology

## Abstract

We present the first report of a specific type of developmental dyslexia in Turkish, letter position dyslexia (LPD). LPD affects the encoding of letter positions, leading to letter migrations within words. In a multiple case study of 24 Turkish-speaking children with developmental LPD, we examined in detail the characteristics of this dyslexia and explored its manifestation in Turkish. We used migratable words, in which a migration creates another existing word (e.g., signer-singer), which exposed the migration errors of the participants. In sharp contrast with the common assumption that dyslexics in transparent languages, including Turkish, do not make reading errors, we have shown that right stimuli can detect even up to 30% reading errors. The participants made migrations in reading aloud, comprehension, lexical decision, and same-different tasks, in both words and non-words. This indicates that their deficit is in the orthographic-visual analysis stage, a stage that precedes the orthographic input lexicon and is shared by the lexical and non-lexical routes. Their repetition of non-words and migratable words was normal, indicating that their phonological output stages are intact. They did not make digit migrations in reading numbers, indicating that the orthographic-visual analyzer deficit is orthographic-specific. The properties of Turkish allowed us to examine two issues that bear on the cognitive model of reading: consonant-consonant transpositions were far more frequent than consonant-vowel and vowel-vowel migrations. This indicates that the orthographic-visual analyzer already classifies letters into consonants and vowels, before or together with letter position encoding. Furthermore, Turkish is very rich morphologically, which has allowed us to examine the effect of the morphological structure of the target word on migrations. We found that there was no morphological effect on migrations: morphologically complex words did not yield more (nor fewer) migrations than monomorphemic ones, migrations crossed morpheme boundaries and did not preserve the morphological structure of the target word. This suggests that morphological analysis follows the letter-position encoding stage.

## Introduction

Dyslexia is a general term relating to a deficit in reading. By now, more than 20 different types of dyslexia have been reported, each with different error types and different characteristics, each resulting from impairments in different stages of the word reading process (Marshall, 1984; Castles and Coltheart, 1993; Ellis and Young, 1996; Jackson and Coltheart, 2001; Castles, 2006; Coltheart and Kohnen, 2012; Castles et al., 2014; Friedmann and Haddad-Hanna, 2014; Hanley, 2017; Friedmann and Coltheart, 2018). Even though much work has been done about types of dyslexia in various languages, almost no studies have examined the way different types of dyslexia manifest themselves in Turkish. In fact, only one paper reported a specific type of dyslexia in Turkish, and it was a study of acquired dyslexia (Raman and Weekes, 2005); we could not find any study that described specific types of developmental dyslexia in Turkish.

In this study, we describe, for the first time, a specific type of developmental dyslexia in Turkish and its characteristics. We report on a multiple case study of 24 Turkish-speaking children who show a developmental dyslexia that affects their ability to encode the position of letters: letter position dyslexia (LPD).

According to the dual-route model (Coltheart, 1981, 1985, 1987; Patterson, 1981; Ellis et al., 1987; Shallice, 1988; Humphreys et al., 1990; Coltheart et al., 1993, 2001; Ellis and Young, 1996), the early stage of word reading includes the visual analysis of the letter string. It encodes the abstract identity of the letters and their relative positions (Coltheart, 1981; Humphreys et al., 1990; Ellis and Young, 1996). Letter position dyslexia is a deficit in the function that encodes the relative positions of letters within words, which leads to letter migrations within words (e.g., *slime* → *smile, cloud* → *could)*. Studies in several languages found that LPD is mainly manifested in migratable words, i.e., words in which letter migration creates other existing words (such as *flies* and *files*, *slept* and *spelt*). Most of the migration errors affect the middle letters, whereas the first and the last letters are relatively immune to migrations. Errors in migratable words are affected by frequency so that more errors occur when the target word (*spelt*) is less frequent than its migration counterpart (*slept*) (Friedmann and Rahamim, 2007). Individuals with LPD were found to make significantly more migrations that involve only consonants than migrations of a vowel and a consonant (Khentov-Kraus and Friedmann, 2018).

Letter position dyslexia has been reported so far for Hebrew, Arabic, and English (Friedmann and Gvion, 2001, 2005; Friedmann and Rahamim, 2007, 2014; Friedmann et al., 2010; Friedmann and Haddad-Hanna, 2012, 2014; Kohnen et al., 2012), in both acquired form (Friedmann and Gvion, 2001, 2005, for Hebrew; and Friedmann and Haddad-Hanna, 2012 for Arabic) and developmental form (Friedmann and Gvion, 2005; Friedmann and Rahamim, 2007; Friedmann et al., 2010 for Hebrew; Friedmann and Haddad-Hanna, 2012, 2014, for Arabic; Kohnen et al., 2012 for English). Until now, LPD has not been reported for Turkish.

### A Brief Description of the Characteristics of the Turkish Language and Orthography

Turkish is morphologically very rich, and a single Turkish word may include multiple suffixes (e.g., the word “*güldüremediklerimizdensin,”* which means “you are the one that we were unable to make laugh,” contains eight suffixes). The modern orthography of Turkish is composed of a 29-letter alphabet of eight vowels and 21 consonants, based on a modified Latin script. In most cases, a single phoneme is represented with a single grapheme, and the grapheme-to-phoneme correspondence is consistent and transparent (Raman, 1999). The only exceptions are words borrowed from other languages, which are usually transferred into Turkish with their original phonology. For example, the word “katip,” which is borrowed from Arabic, is written with a single *a* but read, like in the Arabic origin, with a long *a,/*kaatip/. Syllables in Turkish (except for loan words) comprise of a single vowel. Canonical syllable structure in Turkish is CV, but other structures (such as V, VC, and CVC) also exist. Syllables with consonant clusters are rare (Raman, 1999), and the length of the vowel restricts the consonants of the coda (Kabak and Vogel, 2001). Stress position is regular and final (with some exceptions, Kabak and Vogel, 2001).

### Dyslexia in Turkish

Very few studies about Turkish took the neuropsychological approach to acquired and developmental dyslexia. Or, in Raman and Weekes (2008) more positive words, “Research addressing the cognitive neuropsychology of acquired language disorders in Turkish has only just begun to flourish.” The exception is a study by Raman and Weekes (2005) who studied in detail a Turkish-English bilingual stroke patient who had an acquired lexical-phonological retrieval deficit, which made him unable to read via the lexical route. This led to surface dyslexia in English, and imageability effects in reading Turkish, with good reading of non-words.

We could not find any study on types of developmental dyslexia in Turkish. The few papers that examined developmental dyslexia in Turkish did not make the distinctions between different types of developmental dyslexia and have not characterized the types of errors each of the individuals with developmental dyslexia made. Raman (2011) tested a group of students with developmental dyslexia (without identifying the type of dyslexia each of them had) and examined the effect of Age of Acquisition in word and picture naming in this group in comparison to a non-dyslexic control group. Other studies worked under the assumption that dyslexia in Turkish mainly affects reading fluency (Erden et al., 2002; Özmen, 2005; Ergül, 2012). Studies of reading in typical development focus mainly on the contribution of phonological abilities to reading and spelling acquisition both in monolingual (Öney and Durgunoğlu, 1997; Durgunoğlu, 2002; Kesikçi and Amado, 2005; Babayiğit and Stainthorp, 2010) and bilingual populations (Özdemir et al., 2012; Özata et al., 2016).

The current study, therefore, aims to start filling the gap by (1) reporting and exploring in detail a type of dyslexia that has not been reported for Turkish yet, in either the acquired or developmental form. Exploring its characteristic error types and the properties of stimuli that are more susceptible to errors. (2) Reporting for the first time a specific type of developmental dyslexia in Turkish. (3) Examining the common belief that Turkish readers with dyslexia do not make reading errors – we will demonstrate that, when the relevant stimuli are selected, they definitely do make errors. The rich morphology and the CV structure of Turkish would further allow us to ask questions about properties of LPD that have not been tested so far: (4) What is the relative order of morphological decomposition and letter position encoding. (5) How early in the reading process are letters encoded as consonants or vowel letters.

## Materials and Methods

### Participants

#### Dyslexic Group

The dyslexic participants in this study were 24 monolingual Turkish-speaking children in 4th grade, aged 9–10, 8 males and 16 females. All of them were right-handed. All of the participants were living in Eskişehir, Turkey. They were pupils in regular schools and regular classes. According to the reports of their parents and/or teachers, and through informal observation made by SLPs, none of them had speech and language disorders (beyond reading deficits), nor any history of brain lesion, neurological condition, or cognitive problems. None of them had been previously diagnosed as having dyslexia or learning disability. However, when we discussed their reading with their teachers, the teachers expressed concerns about the reading of almost all the participants.

To select individuals who have developmental LPD for this study, we administered the dyslexia screening task FRİGÜ (Güven and Friedmann, 2014), described in the next section. We included individuals in the LPD group according to the following inclusion criteria: significantly higher total number of errors in the screening test compared with the age-matched group, significantly more letter position errors in the screening test than the control group (and at least 6 letter position errors), and less than 10% for other types of errors.

The 24 children with LPD were identified in the following way: 16 children were identified in a school-wide reading testing in which we administered the FRİGÜ reading screening test to 299 children. The other 8 children were recruited from teachers in 6 schools (in which children from varying SES – low-middle-high– status). The teachers referred children to us who they suspected had learning or reading difficulties. We tested the reading of these children using the FRİGÜ screening test, 8 had LPD and fitted the inclusion and exclusion criteria so they were included in the study.

#### Control Groups

The control group for the screening task included 205 fourth graders, 111 girls and 94 boys, with no report of reading disability. The control group for the further LPD tests included 71 fourth graders aged 9 to 10 years, 39 males and 32 females who had no speech, language, hearing or other cognitive problems, based on teacher or parent reports. Additionally, the children who were examined for the control group were tested by speech-language pathologists, who reported their clinical opinion regarding each child’s language. We excluded three children who the testers suspected to have a language disorder.^1^

### Procedure

Each of the participants was tested individually in a quiet room in the school. All stimuli were displayed on a white page in 14 pt. font, with double vertical spacing between words. No time limit was imposed during testing, the written word lists remained in front of the participants for as long as they needed, and no response-contingent feedback was given by the experimenter. In the silent reading tasks, we instructed the participant not to read aloud. In orally presented tests, the experimenter repeated every item as many times as the participant requested. Each of the participants took part in 13 tests, which were administered in several sessions. The number of sessions and length of each session were determined by each of the participants. The research was approved by the Ethical Committee of Anadolu University, Eskişehir, Turkey. The parents of each child signed written informed consent.

### General Error Coding and Analysis

In the analysis of letter transpositions, we classified the transpositions according to the letters that participated in the transposition: consonant-consonant, vowel-consonant, and vowel-vowel migrations. If the participant produced a sequence of responses to a target word, and one of these responses was an error, we counted the item as being incorrect and analyzed the erroneous response.

### Statistical Analyses

We examined whether each participant with dyslexia performed significantly below his or her age-matched control group using one-tailed Crawford and Howell’s (1998) *t*-test. Within-participant comparisons between two conditions were conducted using chi-squared tests (two-tailed comparisons). At the group level, comparisons between two conditions were conducted using the Wilcoxon Signed-Rank test, which is the non-parametric counterpart of paired samples *t*-test (reported with z statistic), and more than two conditions were compared using Friedman Test. For the correlation, Spearman’s rank correlation coefficient analysis was used. Effect sizes for *t*-tests are reported with Hedges’ *g*, and for Wilcoxon’s, when there is no normal distribution, with *r* (Fritz et al., 2012). Comparisons at the group level between the LPD group and the large control group were done using the *t*-test. An alpha level of 0.05 was used in all comparisons.

## The Screening Test Used to Identify Children With Lpd for This Study

The first test we administered, which we used for initial assessment and identification of individuals with LPD to include in the study, and to examine the properties of their errors, was the screening test from the FRİGÜ test battery (Güven and Friedmann, 2014), which was developed to identify types of dyslexia in Turkish. The screening part of the FRİGÜ is an oral reading test that includes three blocks: 151 single words (2–8 letters long, *M* = 5.12, *SD* = 1.29), 60 word pairs (4–5 letters long, *M* = 4.88, *SD* = 0.92), and 40 non-words (2–9 letters long, *M* = 5.16, *SD* = 1.62). The word reading block was used to identify individuals with LPD according to the criteria described above.

Some researchers have claimed that in certain languages (e.g., languages with transparent orthographies), dyslexia does not manifest itself in errors, only in slow reading. We think this is a misconception that partly results from not using the right stimuli to elicit reading errors. Our approach was, on the basis of the approach of the Tiltan reading battery (Friedmann and Gvion, 2003), to base the reading test on knowledge of the types of words and non-words that are most sensitive to each type of dyslexia, i.e., the type of stimuli in which individuals with this kind of dyslexia make most errors of the relevant type. Ever since the early days of the cognitive neuropsychological approach to dyslexia – [opetwcite]B42,B7[clotwcite]Marshall and Newcombe (1973); Coltheart (1981), and Patterson (1981) found that dyslexias differ with respect to the types of stimuli that are most difficult in them. So there are “dimensions of words” as Patterson called it, for example, surface dyslexia is most evident in reading irregular words, phonological dyslexia in non-words, and deep dyslexia in abstract words, function words, and morphologically complex words.

The word and non-word lists of the FRİGÜ screening test were thus constructed so that they include items that are sensitive to each of the currently known types of dyslexia; words with different stress patterns or with ambiguous grapheme-to-phoneme conversion for identifying surface dyslexia; function words and morphologically complex words to identify phonological output buffer dyslexia, orthographic input buffer dyslexia, and deep dyslexia; abstract words for deep dyslexia; words (and non-words) with many orthographic neighbors for identifying orthographic analyser-output visual dyslexia, orthographic input buffer dyslexia, and letter identity dyslexia; words (and non-words) that can be read as other words by neglecting one side of the word, for identifying neglect dyslexia; and words in which vowel letter omissions, additions, migrations, or substitutions create other existing words, for the identification of vowel letter dyslexia.

The non-words were included for identifying phonological and deep dyslexia as well as various peripheral dyslexias; the word pairs were constructed such that between-word migrations create other existing words, to enable the detection of attentional dyslexia.

Importantly, the screening part of FRİGÜ was designed to also detect LPD. The list of 151 words contains 121 migratable words: 91 words in which a middle letter migration would create another existing word, and 54 words in which migration of exterior letters creates a word (24 of these words allowed for both interior and exterior migrations).

### Results: Reading Screening Test

The participants with LPD made between 6 and 19 letter position errors in the single word reading block, with an average of 10.3 letter position errors (*SD* = 4.1). The control participants, on the other hand, made only 1.8 letter position errors on the average in this task (0–5 errors, *SD* = 2). Each of the LPD children performed significantly poorer than the control group [*t*(204) > 1.94, *p* < 0.02, for each of the participants].

## Oral Reading of Migratable and Non-Migratable Words

Now that the screening task identified 24 children who had LPD, we continued with a line of tests that were developed to examine the nature of this dyslexia, and the way it is manifested in Turkish. We created a list of 183 migratable words to allow for the in-depth assessment of the effect of morphology on migrations (see section “Does Morphological Analysis Precede Letter Position Encoding? Assessing the Interaction of Morphology and Migrations”); the effect of the consonant-vowel status on migration (see section “Is Letter Position Encoding Sensitive to the Consonant-Vowel Status of the Target Letters?”); the position of the migrating letters within the word (assessing middle-exterior, adjacent-non-adjacent, within-across syllable, and length effect, see section “Further Analyses of the Properties of Letter Migrations in Turkish”); and frequency effect (see section “What Is the Nature of the Letter Position Encoding Deficit? Incorrect Underspecified Encoding? The Effect of Frequency on Migrations and Its Theoretical Implication”).

### Experimental Stimuli

The migratable word list included 183 migratable words, 4-to-8 letters long (*M* = 5.2, *SD* = 0.9). Each of these words was such that at least one letter migration within the word results in an existing word (see examples for various types of migrations in Table 1). Each of the words in the list was also such that a letter identity error could create another existing word.

**TABLE 1:** Examples for migration errors of various types that the LPD participants made.

**Condition**	**Target word**	**Response with migration error**	**Translation word**	**Translation response**	**Example from English**
**Middle migrations**					
*Adjacent consonant-consonant (CC) migration*	altı	atlı	six	horseman	badly-baldy
*Adjacent consonant-vowel (CV) migration*	alınma	alnıma	to take offense	to my forehead	from-form
*Consonant-consonant migration across a vowel (C-C)*	ebedi	edebi	eternal	literary	slime-smile
*Vowel-vowel migration across a consonant (V-V)*	çelik	çilek	steel	strawberry	toner-tenor
**Exterior migrations^a^**					
*Adjacent consonant-vowel (CV) migration*	atkı	takı	scarf	jewel	acres-cares
*Consonant-consonant migration across a vowel (C-C)*	yakın	yanık	close	burn	inlet-intel
*Vowel-vowel (Exterior) migration across a consonant (V-V)*	katı	kıta	stiff	continent	demo-dome

*^*a*^There were no words with a potential of exterior adjacent CC migrations, due to the Turkish syllable structure.*

### Results

#### Migrations in Reading Migratable Words

**FIGURE 1:**
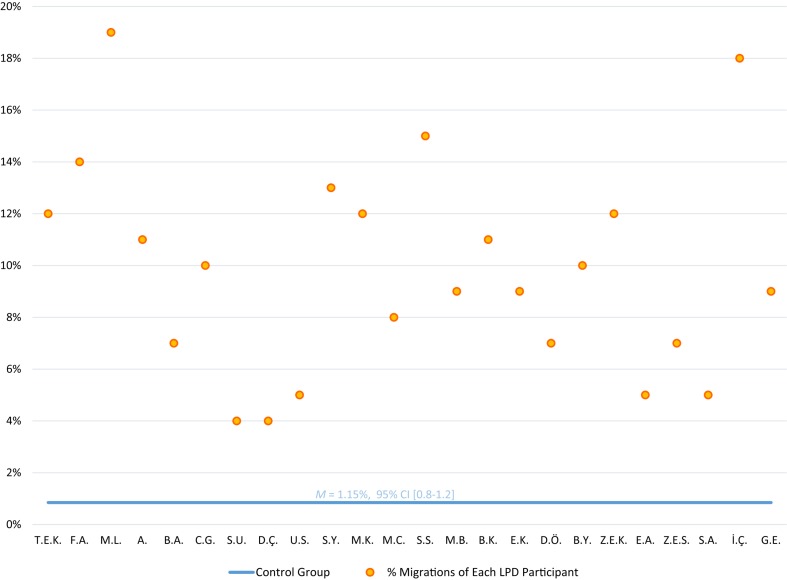
Reading migratable words: % letter position errors of each LPD participant (orange circles) compared to the control group average (horizontal blue line) in reading the list of 183 migratable words.

The LPD participants made a total of 433 migration errors in reading the migratable word list. Figure 1 summarizes the letter position error rates the children with LPD made. Each of the 24 children with LPD made significantly more migrations than the control group (*p* < 0.001, using Crawford and Howell’s, 1998, *t*-test for the comparison of an individual to a control group). The difference was also significant at the group level, where the LPD group made significantly more letter migrations (10% migrations) than the control group (who made only 1% migrations on this list, *SD* = 1.27), *t*(24) = 10.71, *p* < 0.0001, with a very large effect size (*g* = 2.53). See Table 1 for examples for the various types of migrations that the participants with LPD made.

#### Refuting a Misconception: Turkish Dyslexics Do Make Errors When the Appropriate Stimuli Are Presented: Migratable vs. Non-migratable Words

We asked whether the participants make more migration errors when the target words are migratable, i.e., words in which a migration error can create another existing word (like the English word *form*, in which a migration error can create *from*) than on non-migratable words.

We compared the migrations in the list of 183 migratable words to the rate of migrations in reading a list of non-migratable words, which included words in which no single transposition created an existing word (e.g., the target word *gözlük* [glasses], for which all possible migrations result in non-lexical responses). This non-migratable word list included 32 words, 4-to-7 letters long (*M* = 4.9, *SD* = 0.7), with a relatively high frequency (all were among the 5000 most frequent words in Turkish, and more than half of them were among the top 2000 most frequent words, according to Aksan et al. (2016) frequency data.

This analysis showed a striking difference between migratable and non-migratable words: the children with LPD made no migrations in non-migratable words, whereas they made an average of 10% migrations in reading the migratable words (Table 2).

**TABLE 2:** % migration errors in migratable and non-migratable words.

**Participant**	**Migratable words (*N* = 183)**	**Non-migratable words (*N* = 32)**
T.E.K.	12	0
F.A.	14	0
M.L.	19	0
A.C.	11	0
B.A.	7	0
C.G.	10	0
S.U.	4	0
D.Ç.	4	0
U.S.	5	0
S.Y.	13	0
M.K.	12	0
M.C.	8	0
S.S.	15	0
M.B.	9	0
B.K.	11	0
E.K.	9	0
D.Ö.	7	0
B.Y.	10	0
Z.E.K.	12	0
E.A.	5	0
Z.E.S.	7	0
S.A.	5	0
İ.Ç.	18	0
G.E.	9	0
**Average (*SD*)**	**10 (4)**	**0**

*N = number of words that allow for at least one error of the relevant type.*

Relatedly, to examine whether the letter position errors of the participants with LPD tended to create existing words, we analyzed their migration responses in reading migratable words. For each migratable word, there was at least one option for a migration that yields an existing word, and at least one other option for migration response that yields a non-word. We examined whether the participants’ migrations tended to create existing words.

This analysis showed that when they read a migratable word with a migration error, most of their responses were lexical (76.6% of their migration responses were lexical, *SD* = 31.2%). The same was true in reading migratable non-words, in which, as we report below in Section “What Is the Locus of LPD in the Reading Model: Nonword Reading and Silent Reading Tasks – Results”, most errors were lexical as well.

The tendency to produce lexical responses guided us in the analyses of the characteristics of the participants’ migrations: for each analysis, we calculated the rate of errors out of the number of target words in which such errors would create an existing word, i.e., words that have a lexical potential for the relevant error type (for example, we calculated adjacent migrations not out of all words, but rather only out of the target words in which a migration of adjacent letters creates an existing word).

## Does Morphological Analysis Precede Letter Position Encoding? Assessing the Interaction of Morphology and Migrations

Turkish has a very rich morphology, so studying LPD in Turkish readers allowed us to examine a theoretical question about the interaction between letter position encoding and morphological analysis. Specifically, we were interested in the relative order in which letter position encoding and morphological analysis take place. If morphological analysis precedes letter position encoding, then the morphological structure of the target word should affect letter position errors, and migrations should occur only within a morpheme. If, however, letter position encoding precedes morphological analysis, then the morphological structure of the target word should not affect migration errors.

To examine this question, we used three kinds of analysis. We examined whether morphologically complex words yielded a different rate of letter position errors than mono-morphemic words. We also tested whether letter migrations changed the morphological structure of the target word and whether migrations occurred only within morpheme or also across morphemes.

### Analyses and Results

The first analysis examined whether morphologically complex words yield a different rate of letter position errors than morphologically simple words. For this analysis, we compared 44 morphologically-complex words from the migratable word list (which includes words with derivational and words with inflectional morphemes) to 78 monomorphemic migratable words. We selected these words so that they would be matched on length, which led us to include 4–6 letters long morphologically complex words (*M* = 5.39, *SD* = 0.61) and 5–7 letters long monomorphemic words (*M* = 5.22, *SD* = 0.47), so the word lengths in the two groups did not differ significantly.

The results were such that the children with LPD had very similar rates of migrations on the morphologically complex words (8.9%) and on the morphologically simple words (9.5%), and this difference was not significant (*Wilcoxon z* = 0.24, *p* = 0.81).

In the second analysis, we examined whether the migrations changed the morphological structure of the target word. This analysis indicated that there were quite a few migrations that changed the morphological structure of the target word: 21% of the migrations changed the morphological structure of the target word (*SD* = 19%). For example, the monomorphemic target word *akran* (peer) was read with a transposition as “arkan”, a morphologically complex word, constructed from *arka* (back), and the suffix *–n* (singular 2nd person possessor). Or the morphologically simple target word *eskiz* (sketch), which was read with a transposition as the morphologically complex “eksiz” in which the stem *ek* means “supplement” (or, to confuse us, “a morpheme”), and the suffix *siz* means “without” (so this morphologically complex word actually means “without a morpheme” i.e., a monomorphemic word).

The final analysis tested whether migrations occurred only within-morpheme or also across morphemes. This analysis indicated that, in reading the morphologically complex words, the participants transposed letters of the stem with letters of the non-stem morpheme (e.g., *konulu*: konu-lu, themed →*kolunu*: kolu-nu, your arm). Such cross-morpheme migrations occurred on 6.3% of the morphologically complex words (*SD* = 3.7). In fact, migrations within the stem, which involved two letters of the stem (*yenile*: yeni-le, renew → *yinele:* yine-le, repeat) and migrations within the non-stem morpheme (*kirli*: kir-li, dirty → *kiril*, Cyrillic) occurred less frequently than across-morpheme migrations (within the stem: 2% of the morphologically complex words, *SD* = 2.3, within the non-stem morpheme: 0.7%, *SD* = 1.1).

These three kinds of evidence for the lack of sensitivity of letter position errors to the morphological structure of the target word suggest that LPD affects a stage that precedes morphological analysis, and hence, it is not sensitive to the morphological structure of the target words.

## Is Letter Position Encoding Sensitive to the Consonant-Vowel Status of the Target Letters?

To examine the theoretical question of when the consonant-vowel distinction becomes accessible during the process of single word reading, we examined the effect of the consonant-vowel status of a letter on the rate of migrations.

We asked two main questions: whether consonant and vowel letters are differentially susceptible to migrations, and whether they tend to migrate more within their class (consonants transpose with consonants and vowels with vowels) than across class.

### Analyses

For this sake, we compared four types of migration:

(1) V_1_CV_2_: a transposition of two vowels across a consonant [e.g., *eli* (his/her hand) > “ile” (with)]
(2) C_1_VC_2_: a transposition of two consonants across a vowel [e.g., *tak* (attach) > “kat” (floor)]
(3) CC: a transposition of two adjacent consonants: [e.g., *atlı* (horseman) > “altı” (six)]
(4) CV: a transposition of adjacent consonant and vowel [e.g., *atlı* (horseman) > “atıl” (idle)]

We did this analysis only for the participants who did not have vowel dyslexia (Khentov-Kraus and Friedmann, 2018) in addition to LPD, because a higher rate of migrations that involve vowels may, in their case, be a result of their vowel dyslexia.

Within the 183 migratable words list, there were 98 words in which a consonant-consonant migration creates another word (51 words allowing for Adjacent CC migrations and 67 allowing for CC migration across a vowel); 63 words that allow for adjacent consonant-vowel migration, and 74 words that allow for a vowel-vowel transposition across a consonant.

### Results

The results, summarized in Table 3, indicated a clear difference between the different kinds of migration. Migrations that involved only consonants (either adjacent CC transposition or CVC- transposition of two consonants across a vowel) were the most common type of migration (14%), whereas migrations that involved only vowel letters, or migrations that involved a consonant and a vowel occurred less often (5% each). A Friedman’s test indicated that the difference between the three types of migration (C with C, C with V, and V with V) was significant, χ^2^(2) = 30.45, *p* < 0.001, with consonant-only migrations being the most frequent migrations.

**TABLE 3:** Consonant and vowel letter migrations of the participants with LPD in migratable word reading (% migrations of each type out of the number of migratable words with a lexical potential for such error).

**Participant**	**All C-C (*N* = 98)**	**C-C across V (*N* = 67)**	**V-V across C (*N* = 74)**	**Adjacent CC (*N* = 51)**	**Adjacent CV (*N* = 63)**
F.A.	23	16	3	18	6
M.L.	30	18	1	22	17
A.C.	20	13	0	16	5
B.A.	8	4	5	6	5
C.G.	19	13	0	18	2
S.U.	7	4	1	6	2
D.Ç.	5	3	0	4	5
U.S.	8	6	3	8	0
S.Y.	13	10	3	10	14
M.K.	21	22	1	10	2
M.C.	12	12	4	8	0
M.B.	12	6	1	14	8
B.K.	16	10	3	18	3
E.K.	11	6	4	12	5
D.Ö.	6	3	0	4	14
B.Y.	13	6	5	18	3
Z.E.K.	16	12	4	16	5
E.A.	7	3	1	8	3
Z.E.S.	11	3	1	16	2
S.A.	6	3	1	12	3
İ.Ç.	26	28	0	12	13
G.E.	9	9	9	6	0
**Average (SD)**	**14(7)**	**10(7)**	**2 (2)**	**12(5)**	**5(5)**

**Participants with LPD and vowel dyslexia**					
S.S.	9	4	12	12	14
T.E.K.	11	7	12	2	11

*C = consonant letter, V = vowel letter, N = number of words in which at least one error of the relevant type creates an existing word (some words allow for more than one type of error: e.g., the target word “istem” can be read with adjacent CC error as *itsem*, or with a C-C error across V as *ismet*. So it is counted once as a word with any C-C migration potential, and once in words with adjacent CC migrations potential, and also once in words with CC across V migrations potential). Due to the syllable structure in Turkish, we had no words with adjacent VV migration potential and no non-adjacent CV.*

Consonants migrated across vowels (C_1_ V C_2_ → C_2_ V C_1_) significantly more frequently than vowels across a consonant (V_1_ C V_2_ → V_2_ C V_1_), *Wilcoxon z* = 4.42, *p* < 0.0001, *r* = 0.64. Consonants transposed with an adjacent consonant (CC) significantly more often than with adjacent vowels (CV), *Wilcoxon z* = 3.85, *p* < 0.0001, *r* = 0.56.

Another type of analysis that points in exactly the same direction is the analysis of the “preferred migration type” in target words that allow for several types of migrations (C-C, V-V, V-C). There were 64 such target words, and the participants showed the same tendency toward consonant migrations: when they read a word in which several types of migrations were possible, they most often made a C-C migration (67% of the migrations on these words) and had much fewer V-V migrations (17%) or C-V migrations (16%). The C-C migrations were significantly more frequent than the other migration types, Friedman’s test χ^2^ = 21.62, *p* < 0.001.

The results show clearly that consonant letters are more susceptible to transpositions than vowel letters, and that consonant-only transpositions occur more frequently than transpositions that involve a consonant and a vowel letter. Beyond its bearing on the characterization of LPD, this finding that indicates that the classification of letters to consonants and vowels happens very early in the process of orthographic-visual analysis, before or together with letter position encoding, and that consonant letters are processed separately and differently from vowel letters.^2^ It is also interesting to note that the two children who had vowel dyslexia in addition to letter position dyslexia (SS and TEK, presented in the bottom of Table 3) made more transpositions that involve a vowel than transpositions that include only consonants.

## Further Analyses of the Properties of Letter Migrations in Turkish

### Middle vs. Exterior Letter Migrations

Studies of LPD in Hebrew, Arabic, and English report that individuals with LPD make more migrations that involved only middle letters than migrations that involve an exterior letter (Friedmann and Gvion, 2001; Friedmann and Rahamim, 2007; Friedmann and Haddad-Hanna, 2012, 2014; Kohnen et al., 2012). To examine whether this was also the case for Turkish LPD, we compared the rates of migrations that involved only middle letters and migrations that also involved an exterior (first or final) letter.

The 183 migratable words list included 91 words that have at least one possibility for middle migration, 34 words with a possibility for a migration that involves an exterior letter, and 58 words with a potential for both middle and exterior migrations.

The results, presented in Table 4, show that the Turkish-readers with LPD made both middle letter migrations and exterior letter migrations, but they, like LPD participants in the other languages tested, made significantly more migrations of middle letters (9%) than migrations that involved an exterior letter (6%), *Wilcoxon z* = 2.73, *p* = 0.006, *r* = 0.39.

**TABLE 4:** Percentages migrations of the various kinds that the participants with LPD made in reading migratable words.

**Participant**	**Total (*N* = 183)**	**Adjacent (*N* = 86)**	**Non-adjacent (*N* = 120)**	**Middle (*N* = 149)**	**Exterior (*N* = 90)**	**Across syllable (*N* = 153)**	**Within syllable (*N* = 94)**
T.E.K.	12	10	11	10	8	10	6
F.A.	14	16	10	13	7	14	4
M.L.	19	23	13	15	13	17	10
A.C	11	13	8	10	6	11	3
B.A.	7	7	6	6	4	6	4
C.G.	10	10	8	9	6	9	5
S.U.	4	5	3	5	1	4	2
D.Ç.	4	5	3	2	4	3	3
U.S.	5	5	5	5	2	5	3
S.Y.	13	16	8	11	7	11	6
M.K.	12	7	13	7	12	11	5
M.C.	8	5	9	9	2	8	3
S.S.	15	17	10	12	10	10	12
M.B.	9	14	4	7	7	7	6
B.K.	11	13	8	10	6	8	7
E.K.	9	10	6	9	3	7	6
D.Ö.	7	13	2	4	8	4	7
B.Y.	10	14	6	11	2	10	4
Z.E.K.	12	14	8	13	2	10	6
E.A.	5	7	3	4	3	3	4
Z.E.S.	7	12	2	7	1	7	2
S.A.	5	7	3	5	1	5	2
İ.Ç.	18	17	15	13	16	10	19
G.E.	9	16	2	9	3	9	2
**Average (SD)**	**10 (4)**	**12 (5)**	**7 (4)**	**9 (3)**	**6 (4)**	**8 (4)**	**5 (4)**

*N = number of words that allow for at least one error of the relevant type.*

This predominance of middle migrations can also be seen in another type of analysis that assesses the “preferred migration type” in target words in which both middle and exterior migrations create other existing words. There were 55 such target words, and the participants showed the same tendency toward middle migrations: when they read a word in which both a middle migration and an exterior migration would create existing words, they made almost three times more middle migrations (73%) than exterior ones (27%), a difference that was significant, *Wilcoxon* z = 5.06 *p* < 0.0001.

### Migrations of Adjacent and Non-adjacent Letters

Hebrew readers with LPD make more migrations in adjacent letters than non-adjacent letters. We tested whether this is the case also for Turkish children with LPD.

Within the 183 migratable words list, in 61 words only adjacent letter migrations created other existing words, 95 words allowed only for non-adjacent letter migration, and 27 words had a lexical potential for both adjacent and non-adjacent letter migration.

The results, summarized in Table 4, show that the Turkish LPD participants made significantly more migrations in adjacent letters (12%) than in non-adjacent letters (7%), *Wilcoxon z* = 3.09, *p* = 0.002, *r* = 0.45. However, as we report in Section “Is Letter Position Encoding Sensitive to the Consonant-Vowel Status of the Target Letters?,” the consonant-vowel status of the letter affects migration considerably; once the consonant-vowel status is kept constant (analyzing only consonant-consonant migrations), the size of the difference between adjacent and non-adjacent letters shrinks, *Wilcoxon z* = 2.02, *p* = 0.04.

### Migrations Across and Within Syllables

We also investigated whether more migrations occur within- or across syllables. The results, presented in Table 4, show that there were significantly more across-syllable migrations (8%) than within-syllable migrations (5%), *Wilcoxon z* = 3.07, *p* = 0.002, *r* = 0.44.

### Length Effect

To examine the effect of word length on the rate of migrations, we analyzed the participants’ migration rates in reading aloud the list of 183 4–8 letter migratable words (see section “Experimental Stimuli”).

We conducted Spearman’s Rho correlation coefficient analysis to see if there is a relationship between length and migration errors. A correlation analysis of the results (presented in Table 5) indicated that the correlation coefficient was low and non-significant (*R* = −0.11, *p* = 0.19).

**TABLE 5:** The relationship between the number of letters and LPD error rate.

**Word Length**	**Number of words**	**% migrations**
4 letters	31	10
5 letters	101	9
6 letters	33	8
7 letters^a^	18	19

*^*a*^This group included 17 7-letter words and a single 8-letter word.*

## What Is the Locus of Lpd in the Reading Model: Non-Word Reading and Silent Reading Tasks

The next question was where in the word-reading model is the impairment that gives rise to LPD. For this sake, we examine the participants’ non-word reading and compare it to their word reading (section “Non-word Reading” below): if they make migration errors in non-words as well, this would mean that the deficit is not in lexical components and that it is rather in a component that is shared by lexical and non-lexical processes. We then test the participants’ silent reading using various tasks to examine whether the locus is in the orthographic-visual analysis stage. If it is, a deficit in the orthographic-visual analysis should cause migrations not only in reading aloud but also in lexical decision and comprehension of written words (section “Silent Reading Tasks” below). If the deficit is indeed in the orthographic input, phonological output should not show migrations when the input does not involve reading. This we tested in Section “Assessing Phonological Output Using Non-word and Word Repetition,” which examined the participants’ phonological output using non-word and word repetition.

### Non-word Reading

To examine how the LPD participants read non-words, and to further test whether their deficit was pre-lexical or at a lexical stage, we presented them with an additional list of 60 non-words. The non-words in the list were 4-to-6 letters long (*M* = 4.92, *SD* = 0.42). Half of the non-words (30) were migratable, i.e., non-words in which a letter migration creates an existing word (e.g., the non-word “bakrı” is migratable because the migration of the two middle consonants creates the word “barkı,” his/her home). The other 30 non-words were non-migratable so that no migration created another word (e.g., “solik” or “bikeş”).

The 30 migratable non-words were selected to allow the examination of the characteristics of migrations also in non-words. To compare middle and exterior letter migrations, 27 of the non-words were such that at least one migration of an exterior letter would create an existing word, and 10 non-words were such that migration of middle letters would create an existing word (7 of these words had both middle and exterior migration potential). There were 15 words that had a potential of consonant-consonant transposition, 3 words with a potential of vowel-vowel transposition, and 25 words with vowel-consonant transposition potential.

#### Results

**FIGURE 2:**
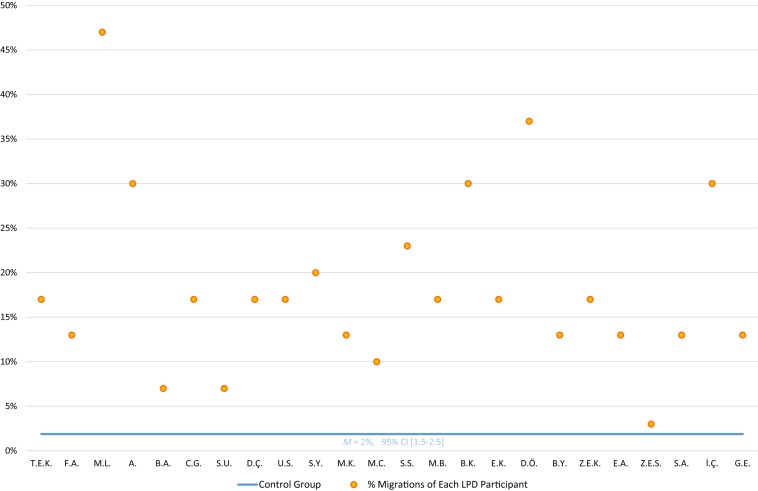
Migratable non-word reading: % letter position errors of each LPD participant orange circles) compared to the control group (horizontal blue line).

The 24 participants with LPD made an average of 18% migrations when reading the migratable non-words. The rate is significantly higher than that of the control group (2%), *t*(25) = 7.63, *p* < 0.0001, *g* = 1.8. On the individual level (see Figure 2), 21 children with LPD made significantly more migration errors than the control group (14 children *p* < 0.001, and 7 children *p* < 0.05, using Crawford and Howell’s (1998), *t*-test).

The participants made significantly more migrations in reading the migratable non-words (18%) than in reading the non-migratable non-words, where they made 7% migrations (*Wilcoxon z* = 4.41, *p* = 0.0001, *r* = 0.64). Still, the rate of migrations that the children with LPD made in non-migratable non-words was significantly larger than that of the control group, who only made 0.3% migrations in non-migratable non-words, *t*(23) = 4.92, *p* < 0.0001, *g* = 1.16. This finding is important because it indicates that the deficit that underlies the migration errors in LPD is not in the orthographic input lexicon but rather in an earlier stage that affects words, non-words, and non-migratable non-words: the orthographic-visual analyzer.

When we analyzed the errors that the LPD participants made in reading the migratable non-words (presented in detail in Table 6), we see that, like in their reading of the existing migratable words, they made significantly more migrations in middle letters (31%) than migrations that involved an exterior letter (9%; *Wilcoxon z* = 4.51, *p* = 0.0001, *r* = 0.65). They made significantly more migrations that involved only consonants (22%) than migrations of a consonant and a vowel (11%), *Wilcoxon z* = 3.01, *p* = 0.001, *r* = 0.44, and significantly more migrations across syllables (24%) than within a syllable (9%), *Wilcoxon z* = 4.30, *p* = 0.0001, *r* = 0.62.

**TABLE 6:** Characteristics of migration errors in reading migratable non-words (% errors in each of the stimulus types).

**Participant**	**Total (*N* = 30)**	**Middle (*N* = 10)**	**Exterior (*N* = 27)**	**Across syllable (*N* = 14)**	**Within syllable (*N* = 25)**	**C-C (*N* = 15)**	**V-V (*N* = 3)**	**C-V (25)**	**Adj. CC (*N* = 13)**	**Adj. CV (*N* = 19)**
T.E.K.	17	10	15	7	16	13	0	16	8	16
F.A.	13	30	4	21	4	20	0	4	23	5
M.L.	47	50	33	36	36	33	0	44	31	47
A.C.	30	60	11	57	4	40	33	12	46	11
B.A.	7	10	4	14	0	7	33	4	8	0
C.G.	17	50	0	36	0	33	0	0	38	0
S.U.	7	20	0	14	0	13	0	0	15	0
D.Ç.	17	50	0	36	0	33	0	0	38	0
U.S.	17	30	7	29	4	13	33	12	15	11
S.Y.	20	30	11	21	12	27	0	12	23	11
M.K.	13	30	4	21	4	27	0	4	23	0
M.C.	10	10	7	14	4	13	33	8	8	0
S.S.	23	40	7	29	12	27	0	16	23	16
M.B.	17	30	7	21	8	27	0	8	31	0
B.K.	30	50	15	43	12	33	67	20	31	11
E.K.	17	40	4	21	8	20	0	8	31	5
D.Ö.	37	30	30	29	28	20	33	32	31	32
B.Y.	13	10	11	7	12	7	0	12	8	16
Z.E.K.	17	10	15	7	16	7	0	16	8	21
E.A.	13	40	0	29	0	27	0	0	31	0
Z.E.S.	3	10	0	7	0	7	0	0	8	0
S.A.	13	20	7	21	4	13	33	8	15	5
İ.Ç.	33	50	19	43	16	47	0	20	38	11
G.E.	13	30	4	21	4	27	0	4	23	0
**Average (SD)**	**18 (10)**	**31 (16)**	**9 (9)**	**24 (13)**	**9 (9)**	**22 (11)**	**11 (19)**	**11 (11)**	**23 (12)**	**9 (12)**

*C = consonant letter, V = vowel letter, N = number of words that allow for at least one error of the relevant type, Adj. = Adjacent.*

Like in the word reading, most of the error responses the LPD participants made in reading the migratable non-words were lexical (*M* = 81.3% of the total errors in migratable non-words, *SD* = 17.1%), with significantly more lexical errors than non-lexical errors, *Wilcoxon z* = 5.36, *p* < 0.001, *r* = 0.77. When we only look at migration responses in reading the migratable non-word list, the picture remains the same: most of their migration responses were lexical (*M* = 88.6% of their transposition errors in migratable non-words, *SD* = 44.3%; significantly more lexical than non-lexical migration responses, *Wilcoxon z* = 5.63, *p* < 0.001, *r* = 0.82).

Not surprisingly, when they made migration errors in reading non-migratable non-words, where migrations could not yield an existing word, they produced mainly non-lexical responses (*M* = 33.6% of the total errors in non-migratable non-words, *SD* = 35.1%, *Wilcoxon z* = 2.87, *p* = 0.002, *r* = 0.42).

### Silent Reading Tasks

If the source of letter migrations is indeed in a deficit in the orthographic-visual analysis stage, we would expect migrations to occur in silent reading tasks that do not involve reading aloud. To examine this, we ran 3 reading tasks that did not involve reading aloud: lexical decision, same-different decision, and comprehension.

#### Lexical Decision

The word list for lexical decision included 59 items: 29 words and 30 non-words, 4–5 letters long (*M* = 4.89, *SD* = 0.37). All the words and non-words were migratable. We asked the participants to read the list silently and mark only the existing words.

##### Results

**FIGURE 3:**
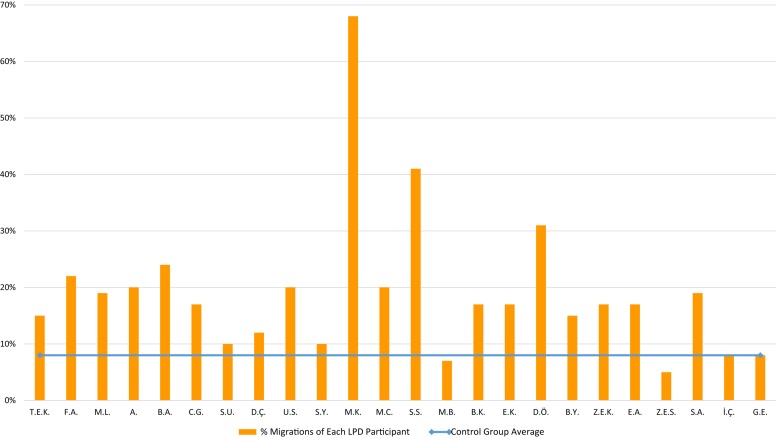
Lexical decision: % letter position errors of each LPD participant (orange columns) compared to the control group average (blue horizontal line).

The participants with LPD made an average of 19% (*SD* = 12.9%) errors on the lexical decision task, significantly more than the control group (8%, *SD* = 5.4%), *t*(25) = 4.06, *p* = 0.0004, *g* = 0.96. The participants made 20% errors of accepting migratable non-words as existing words, and 18% errors of judging existing words as non-words. The individual performance of the LPD participants is presented in Figure 3. In the individual level analysis, 16 of the 24 LPD participants performed significantly poorer than the control group (*p* < 0.05).

#### Same-Different Decision

In the same-different task, the participants were presented with 60 written pairs of 4–7 letter words (*M* = 5.08 letters, *SD* = 0.67), presented side by side with a single space between them. Half of the pairs (30 pairs) included two migratable words that differed in the position of the middle letters. The other 30 pairs included identical migratable words. We asked the participants to decide, for each pair, whether the two words were the same or different.

##### Results

**FIGURE 4:**
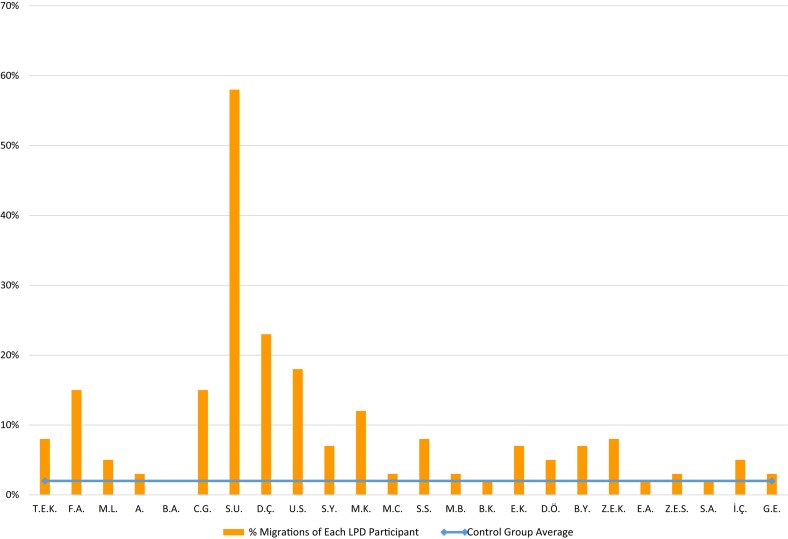
Same-different task: % letter position errors of each LPD participant (orange columns) compared to the control group average (blue horizontal line).

The participants with LPD made significantly more errors in this task (9%) than the control group (who made 2% errors), *t*(23) = 2.83, *p* = 0.009, *g* = 0.67. The LPD participants made 9% errors in which they said “same” for pairs of words that differed in the order of letters, and 10% errors in which they said “different” for identical pairs of migratable words. The analysis of the individual performance of the LPD participants, presented in Figure 4, showed that 12 of the 24 participants performed significantly poorer than the control group (*p* < 0.05).

#### Comprehension Task: Migratable Word Association

We assessed the comprehension of migratable words using a word association task. The task included 28 items. Each item included 4 words: a target migratable word and 3 words from which the participant needed to select one. The target migratable word allowed for at least two different migrations that can create existing words. The three options included one word that is semantically related to the target word (e.g., for the target word *eksi*, minus, the semantically related word was *negatif*, negative). The two distractor words were semantically related to possible migration counterparts of the target word. For example, “eksi” can be read with migration as “eski” (old) and as “kesi” (cut), so for the first migration, we have presented the word “yeni” (new), and for the second migration “bıçak” (knife). We selected target words according to the characteristics that we knew induced more migrations in our participants’ reading: most of the target words had a potential for middle CC migration, and the target words were less frequent than their migration result, which were semantically related to the distractors. We tried as much as possible to use non-migratable words for the three options (88% of the options were non-migratable).

The target word was presented in orange on the left, and the three options were presented in black, one above the other to its right, in random order. We requested the participants to select the word that was most related to the target word.

##### Results

**FIGURE 5:**
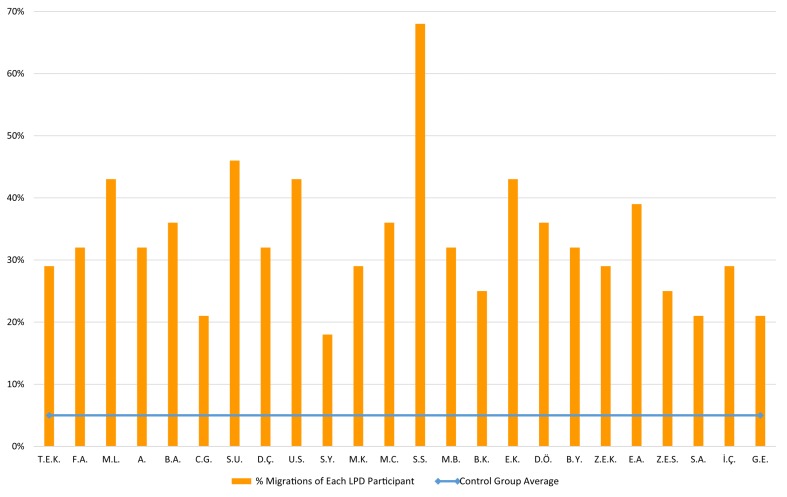
Comprehension task: % letter position errors of each LPD participant (orange columns) compared to the control group average (blue horizontal line).

The children with LPD made 33% errors in this task, an error rate that was significantly higher than that of the control group (which was only 5%), *t*(24) = 9.61, *p* < 0.0001, *g* = 2.27. Each of the LPD participants made significantly more errors in this task than the control group (for 20 LPD children, *p* < 0.001; for the rest 4 children, *p* < 0.05), see Figure 5 for the performance of each participant.

### Assessing Phonological Output Using Non-word and Word Repetition

In order to further explore the locus of impairment that gives rise to LPD and to examine an alternative explanation according to which the migration errors resulted from a deficit in the phonological output buffer, we assessed these children’s phonological output using a non-word repetition task and a task of repeating words that the participant had read with a migration error.

#### Non-word Repetition

The participants repeated non-words using a standardized non-word repetition task (Turkish Non-word Repetition Test, Topbaş et al., 2014). The test included 30 items (1–5 syllables long), which consisted of 15 non-words that violate Turkish phonotactic constraints, 10 non-words that obey Turkish phonotactic rules, and 5 morphologically complex non-words. The test is normed with 150 typically developing children.

##### Results

All of the 16 LPD children who participated in the non-word repetition task performed this task within the normal range, with scores above the threshold for impaired repetition. The mean number of correct repetition of the LPD participants was 27.7 (out of 30), *SD* = 1.47.

#### Migratable Word Repetition

For each of the 19 children who participated in this task, we selected 10 of the migratable words that they read with a migration error and we then asked them to repeat these words.

##### Results

All of the 19 LPD children who participated in the migratable word repetition task performed this task flawlessly, with no migration error, and in fact, with no other error too.

The results of the two repetition tasks indicate that the participants had no phonological output buffer deficit, and support our conclusion, reached on the basis of the silent reading tasks, that the origin of the deficit that underlies LPD is in the input reading stages.

### Theoretical Conclusion: LPD Is a Deficit in the Letter Position Encoding Function in the Orthographic-Visual Analysis Stage

**FIGURE 6:**
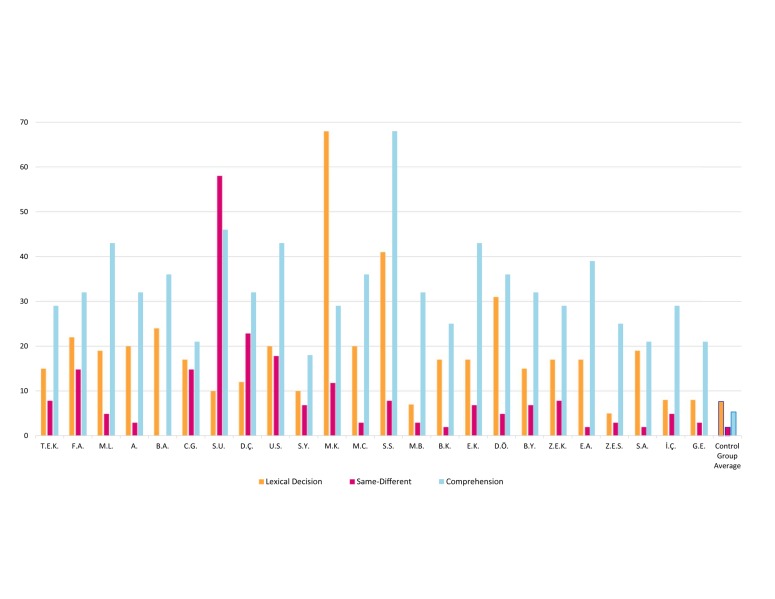
Percentage errors of each of the LPD participants on the three silent reading tasks in comparison to the control group (control data reported in the rightmost columns).

The results of the three silent reading tasks: same-different decision, lexical decision, and written word comprehension (summarized in Figure 6) all point to the same conclusion: LPD affects not only reading aloud but also tasks that involve reading without oral production. These results, together with the findings that LPD affected both words and non-words, point to the locus of impairment in the reading model as a deficit that affects the early pre-lexical stage of orthographic-visual analysis rather than the phonological output stages. This conclusion is supported by the normal phonological output abilities the participants demonstrated in non-word and migratable words repetition.

## What Is the Nature of the Letter Position Encoding Deficit? Incorrect or Underspecified Encoding? the Effect of Frequency on Migrations and Its Theoretical Implication

Examining the effect of the relative frequency of the target word and its migration counterpart can shed light on the nature of the letter position encoding deficit in LPD. Two options are imaginable: incorrect position encoding and underspecified position. If the nature of the letter position deficit is incorrect encoding of letter positions, the word with the incorrect positions is identified in the orthographic input lexicon according to the incorrect information that arrived from the orthographic-visual analyzer and no effect of frequency is expected. If, however, the nature of the letter position encoding deficit is that the position of some (usually middle) letters is not encoded, frequency should affect the error rates. This is because letter position is encoded at a stage before the orthographic input lexicon, and partial position information that is transferred to the orthographic lexicon, which is organized by word frequency, should first activate the more frequent word of the migratable word pair. Thus, according to the partial letter position encoding hypothesis, when the target word is less frequent than its migration counterpart, the less frequent word is expected to be read as the more frequent one; the more frequent target word is expected to be read with fewer migrations.

### Method

In order to examine the effect of frequency on letter migrations in children with LPD, we wanted to use a frequency rating that is appropriate for their age and their familiar world. We, therefore, collected frequency ratings from 30 typically-developing children in the same age and classes as our LPD participants. We presented them with 305 pairs of migratable word pairs – the target word we used in the test and its possible migration result. We asked the children to judge, for each pair, which of the two words was more familiar to them, and occurred more frequently in what they read. We collected their judgments and defined, for each pair, which word was more frequent. Then, we selected the target-response pairs for which there was a clear frequency difference.^3^

### Results

The results indicated that there were far more migrations when the target word was clearly less frequent than the migration result (21.2% migrations on the average) than when the target word was clearly more frequent than its migration result (9.0% migrations on average). This comparison was significant (*Wilcoxon z* = 4.24, *p* < 0.0001, *r* = 0.61).^4^ This indicates that frequency affects migrations and supports the partial position encoding hypothesis.

## Number Reading

To examine whether LPD results from a general visual/perceptual deficit or whether it rather pertains to orthographic material only, we examined the LPD children’s reading of multi-digit numbers. We presented 40 multi-digit numbers (2–4 digits long, *M* = 3.1 digits, *SD* = 0.8), and asked the participants to read each number aloud.

### Results

The LPD participants, who made a considerable rate of migrations in reading words, made very few migration errors when they read numbers. They made only 0.25% migration errors in reading multi-digit numbers aloud. Of the 24 children, 21 made no digit migrations at all in reading numbers, and three children made a single digit migrations error. This migration rate in numbers was significantly smaller than the rate of migrations that the same children made in reading words, *Wilcoxon z* = 6.18, *p* < 0.0001, *r* = 0.89. This remains a significant difference if we only take digit migrations in the 15 4-digit numbers (0.3%) and compare them to letter migrations in the 4–5 letter words (9.5%), *Wilcoxon z* = *6.11*, *p* < 0.0001, *r* = 0.89 (as we have seen above, there is no length effect in migrations in word reading, and the rate of migrations is identical in 4-, 5-, and 6 letter words). The comparison of digit migrations to letter migrations in non-words yielded a similar result: the LPD group made significantly more letter migrations in migratable non-words (*M* = 18.38, *SD* = 0.10) than digit migrations in numbers (*M* = 0.38, *SD* = 0.01), *Wilcoxon z* = 6.15, *p* < 0.0001, *r* = 0.89.

There was no significant difference between the rate of digit migrations in the LPD group (*M* = 0.13, *SD* = 0.33) and in the control group (*M* = 0.22, *SD* = 0.42) (in fact, the LPD group even had a slightly smaller digit migration rate than the controls), *t*(50) = 1.07, *p* = 0.28. As 21 of the LPD participants made no digit migrations at all and 3 LPD participants made a single digit migration, none of the LPD participants differed from the control group in number reading.

## Discussion

This study identified a first specific type of developmental dyslexia in Turkish, developmental LPD. This is the first report of LPD in Turkish, and it joins reports of LPD in Hebrew, Arabic, and English (Friedmann and Gvion, 2001, 2005; Friedmann and Rahamim, 2007, 2014; Friedmann et al., 2010, 2015; Friedmann and Haddad-Hanna, 2012, 2014; Kohnen et al., 2012; Kezilas et al., 2014), enriching our understanding of this dyslexia and its characteristics. It also joins a growing body of evidence showing that not only acquired dyslexia, but developmental dyslexia also has various types (for reviews see Marshall, 1984; Castles and Coltheart, 1993; Castles, 2006; Coltheart and Kohnen, 2012; Castles et al., 2014; Hanley, 2017; Friedmann and Coltheart, 2018).

### Turkish Dyslexics Do Make Errors in Reading Aloud, Once the Appropriate Stimuli Are Presented

It is especially interesting that this dyslexia was found in Turkish, in light of the fact that researchers of dyslexia in Turkish claim that dyslexia only manifests itself in fluency impairments (Erden et al., 2002; Özmen, 2005). In fact, Raman (2011) and Ergül (2012) claimed that Turkish-speaking children with dyslexia read accurately (their accuracy was age-appropriate) but their reading fluency is below the normal level. These suggestions follow a tradition of dyslexia research suggesting that in languages with transparent/consistent orthographies, individuals with dyslexia do not make errors but can only be detected on the basis of their lower reading speed (e.g., Wimmer, 1993).

We believe that the generalization that dyslexic readers of transparent orthographies do not make reading errors is a misconception. First, the transparency of an orthography should only affect the rate of errors in oral reading in cases of surface dyslexia. Namely, individuals with an impairment in the lexical route, who are forced to read words via the sublexical route, are expected to make fewer errors in reading words if the grapheme-to-phoneme conversion is consistent and often provides the correct reading. However, crucially, surface dyslexia is only one of 21 types of dyslexia, and the orthographic depth of a language is not expected to affect the other types of dyslexia. Additionally, different dyslexias yield different types of errors and are affected by different dimensions of words. Therefore, to identify each type of dyslexia, the relevant stimuli need to be presented, otherwise, the person with dyslexia will not make reading errors. Therefore, for example, to detect surface dyslexia, one needs to present irregular words; to detect phonological dyslexia, one needs to present non-words, and to detect deep dyslexia one needs to present function words, abstract words, and morphologically complex words. To detect LPD, one needs to present migratable words, i.e., words in which letter migration creates other existing words. And indeed in this study, we presented to the Turkish reading dyslexics migratable words and non-words and they made migration errors in reading, sometimes even up to 30% of the words (on migratable words that allowed for consonant migration) and to 47% of the migratable non-words.

The participants made far more errors on migratable words (and non-words) than on non-migratable words (and non-words). In fact, they did not make migrations on the non-migratable words. This finding is in line with the lexical tendency that the participants showed in their migration responses: most of their migration responses (in reading the migratable word and the migratable non-words) were lexical. This means that the diagnosis of LPD in Turkish critically hinges on the types of words that are presented to the participant: if migratable words are not presented, the participant’s LPD may be missed.

Through the analyses of these migration errors, the study examined, in detail, the characteristics of LPD and the way it manifested itself in Turkish. We found that many of the characteristics of LPD reported for other languages held also for our Turkish-speaking participants more middle than exterior letter migrations, slightly more adjacent than non-adjacent migrations, and we were also able to discover new properties, which the special nature of the Turkish language and orthography allowed us to examine. Below, we report and discuss the main properties of LPD in Turkish that emerged from this study.

### No Effect of the Morphological Structure of the Word

Turkish has very rich morphology, which made it a wonderful testing ground for examining the effect of morphology on letter position encoding. We examined several points regarding the interaction of morphology and letter position encoding. First, we asked whether more migrations occurred in morphologically complex words compared with morphologically simple ones. The results were that there were no differences between the rates of migrations in morphologically complex and morphologically simple words.^5^ In addition, many of the migrations changed the morphological structure of the target word. The migrations were also insensitive to whether the letters belonged to the stem or the affix: the participants actually made more transpositions of letters of the stem with letters from the non-stem morpheme than within-morpheme migrations.

These findings suggest, in line with Friedmann et al. (2015), that letter position encoding precedes morphological analysis, a conclusion that is quite sensible: to perform morphological analysis, the system needs to know first exactly where each letter is localized.

### Migrations Are Sensitive to the Consonant-Vowel Status of the Migrating Letters

In a recent paper, Khentov-Kraus and Friedmann (2018) reported on vowel dyslexia, which selectively affects the reading of vowel letters. This dyslexia results from a selective deficit in the processing of vowel letters in the sublexical route. In the framework of that paper, the researchers also analyzed migration errors of 48 Hebrew readers with LPD and found that they make more errors that involve the transposition of two consonants than transpositions of a vowel and a consonant. This, in turn, was taken to suggest that the orthographic-visual analyzer is already sensitive to the consonant-vowel status of the target letters. In the current study we took this examination one step further, by using the opportunities offered to us by the Turkish language and orthography. We compared CC transpositions (transpositions of a consonant letter with another consonant letter), with CV transpositions (transpositions of a consonant letter with a vowel letter), and added a comparison that has not been done yet, of VV transpositions – transpositions that only involve two vowels exchanging positions. We did so by selecting three types of migratable words, each allowing different types of transposition.

The results were clear-cut: CC migrations occurred almost three times more than either VC or VV migrations. This finding applies also to non-words, where most of the migrations involved consonants only. This finding has a very important bearing on the orthographic-visual analyzer: it means that already at the stage of letter position encoding, the orthographic-visual analyzer is sensitive to the consonant-vowel status of the letter. Namely, even though *consonant* and *vowel* are phonological notions, the orthographic processing is sensitive to this distinction at the letter level, and distinguishes between consonant letters and vowel letters already at the orthographic-visual analysis stage, long before the phonological stages of reading.

This difference between migrations of consonant and vowels also says something about the nature of the LPD deficit: it is not a visual deficit, but rather a deficit in a later, orthographic stage. Had the deficit been visual, no difference between consonant and vowel letters would be predicted.

The finding that there were mainly CC migrations also accounts for the finding that more migrations occurred between syllables than within a syllable: syllable structure in Turkish is regular, and syllables take the forms CV, VC, CVC, and VCV. As a result, migration within a CV or VC syllable will always be CV migration, whereas migration across syllables can be CC migration. The tendency to make more CC migrations results in making more across-syllables migrations.

### The Locus of the Deficit That Gives Rise to LPD

#### The Deficit Is in a Stage Shared by the Lexical and Non-lexical Routes: Non-word Reading

We tested the participants’ reading, not only of existing words but also of non-words. This is important in order to examine whether the deficit indeed lies in the orthographic-visual analyzer or whether it stems from a deficit in the orthographic input lexicon. We found that the participants made migration errors not only on words but also on (migratable and non-migratable) non-words, and that their non-word reading shows the same error types (migrations) and the same characteristics as word reading. These findings indicate that the deficit that gives rise to LPD has to reside in a non-lexical stage that is shared by the lexical and sublexical routes.

#### The Deficit Is in an Input Reading Stage and Not in a Phonological Output Stage

Two such shared stages exist the orthographic-visual analyzer and the phonological output buffer. Which of them is responsible for LPD? If the deficit lies in the orthographic-visual analyzer, then the deficit should not only affect reading aloud but also other tasks that involve reading input, even without oral production. If the deficit is in the phonological output buffer, silent reading tasks should not involve migrations. To examine this, we tested the participants’ same-different decision, lexical decision of migratable non-words, and the comprehension of migratable words that required distinguishing between the target word and its migration counterpart.

We found that all the LPD participants had letter migrations not only in oral reading but also in at least one of these silent reading tasks. These results support the localization of the deficit in the orthographic-visual analysis, pre-lexical stage.

To further explore this point, and examine the phonological output buffer stage, we also asked them to repeat the migratable words they had read with a migration error. Had the deficit originated in the phonological output stage, we would expect the participants to also make these errors when they repeated the same words. The results unequivocally showed that they were unimpaired in the phonological output stage – they repeated the migratable words correctly, and significantly better than their reading of the same words. We reached the same conclusion on the basis of a non-word repetition task in which these participants performed within the normal range, again, ruling out a deficit in their phonological output stage. Additionally, the finding that there was no significant length effect on migrations also supports the conclusion that the deficit does not reside in the phonological output buffer.

To conclude, then, the results indicate that the participants’ deficit that gives rise to LPD is in the orthographic-visual analyzer, in the function responsible for letter position encoding. This function is already sensitive to the consonant-vowel status of the target letters but is not sensitive to the morphological structure of the target word.

### Letter Positions Are Underspecified, Rather Than Incorrectly Encoded: Evidence From the Frequency Effect

The frequency had a significant effect on migrations. The participants made far more migrations when the target word was less frequent than the migration result than when the target word was more frequent than its migration result. This, too, has implications for diagnosis, as well as for the description of the LPD impairment. Clinically, it means that in order to detect LPD, it is better to present the less frequent migratable word than its more frequent counterpart. Theoretically, it provides insights as to the nature of the impaired process in LPD.

One can imagine two possibilities for the failure in letter position encoding: one is that letter identities are bound to incorrect letter positions, the other is that the position of some (usually middle) letters is not encoded. These two descriptions bear different predictions with respect to the effect of frequency on migrations: if it is erroneous letter position encoding, the input to the orthographic input lexicon is letters that appear in an incorrect order, and if this letter order exists in the lexicon, it doesn’t matter how frequent it is, so we would not expect frequency to affect the errors. If, on the other hand, frequency does have an effect, as we see here, it means that letter positions are not encoded and then the lexicon is searched with this partial information, of letter identities without positions. In this case, the orthographic input lexicon finds the first lexical entry that matches the partial information, which will usually be the more frequent word. Thus, the frequency effect we detected suggests that our participants did not encode the position of some of the letters, rather than encoded it incorrectly.

### Not a General Deficit in Sequence Perception: Normal Number Reading

Another question that is often raised with respect to LPD is whether it is dyslexia that affects only orthographic material or whether it is a more general perceptual deficit that also affects other sequences. To examine this, we tested our participants’ reading of multi-digit numbers. We found that none of the participants had a deficit in reading numbers and none of them made more digit migrations than the controls.

This indicates, in line with other studies on LPD (Friedmann et al., 2010) and on other dyslexias in the orthographic-visual analyzer (see Dotan and Friedmann, 2019, for a review of dissociations between dyslexia and dysnumeria), that the orthographic-visual analyzer is orthographic-specific and does not handle digits. It further indicates that LPD is orthographic-specific.

### Theoretical Implications for the Reading Model

These results bear theoretical implications for the word reading process. Firstly, the finding that letter migrations were unaffected by the morphological structure of the target word suggests an insight with respect to the relative order of letter position encoding and morphological analysis. It indicates that letter position encoding happens before the system can parse the morphological structure of the target word. This makes sense, as morphological analysis needs to apply to strings of letters that are already bound to positions within the word.

A second theoretical implication regards the processing of consonant and vowel letters. The findings that consonant transposed with other consonants far more often than consonants with vowels, and that consonants migrated more than vowels, indicate that the consonant-vowel status of the letter is already computed early in the orthographic-visual analysis stage, before letter position encoding. This finding also suggests that the position of consonant and vowel letters is encoded separately. The finding that consonant-consonant migrations were far more frequent than consonant-vowel migrations, which was also found in Khentov-Kraus and Friedmann (2018) for Hebrew, can be accounted for by assuming that consonant letters and vowel letters are encoded in two separate layers – a consonant-letters layer and a vowel-letters layer, in which the letters are ordered by their position. If we assume that migrations occur more readily within a layer, this would account for more consonant-consonant than consonant-vowel migrations. However, the new finding from Turkish LPD, that there are also more consonant-consonant migrations than vowel-vowel migrations suggests that the position of consonant letters and of vowel letters is encoded not only separately, but differently. The view should probably not be that of two separate layers of consonants and vowels, with migrations occurring mainly layer-internally. It possibly suggests that the position of the consonants in the word is computed first, creating an ordered consonantal skeleton, and then each vowel letter is inserted into the consonant skeleton. Under such mechanism, LPD mainly affects the position encoding of the consonants in the consonantal skeleton.

Finally, as we summarize above, the selective position-encoding impairment, which affected letters but not digits, indicates that the orthographic-visual analyzer is orthographic-specific and does not handle digits (Friedmann et al., 2010; Dotan and Friedmann, 2019).

### Clinical Implications

Research on Turkish often refers to fluency as the only reading aspect that is impaired in dyslexia, and possibly, as a result, dyslexia studies only report fluency measures. Some researchers conclude that Turkish readers with dyslexia do not make more errors than controls (e.g., Raman, 2011). This study showed that it is both possible and essential to also look at children’s errors. To expose reading errors, it is crucial to present stimuli that will be sensitive to each type of dyslexia and will induce the relevant errors from the readers. In our case, it was migratable words that were presented and revealed that Turkish readers with dyslexia *do* make reading errors, once the appropriate stimuli are presented to them. Our study shows that, in order to diagnose LPD, the toolkit for diagnosis has to include migratable words. We were able to identify this dyslexia because we used the FRİGÜ screening test, which we created to be sensitive to the various types of dyslexia. To identify LPD, we included in the test 121 migratable words and 22 migratable non-words. These stimuli exposed the LPD of our participants. In contrast, the non-migratable words did not yield migrations. This means that if we only used non-migratable words for testing, we would have missed the source of reading difficulty of our subjects.

Once the right stimuli are presented, it becomes possible to diagnose persons with dyslexia not only on the basis of their reading speed but also based on their error rates and the types of errors that they make. This would be a way to explain to the person with dyslexia what their problem is and to start targeting treatment at the impaired components.

And in fact, slow reading is not as detrimental to reading as are errors in reading. The parents who came with their children for the reading tests reported to us only the fact that the children were not reading correctly, and their concerns were about their children making errors, in reading aloud and also in understanding what they read. This applied more generally, not only for the parents of children who we eventually found to have LPD but also for children with surface dyslexia, attentional dyslexia, and vowel dyslexia.

A further clinical conclusion related to the properties of the migratable words selected for the diagnostic word list: in order to trigger more errors, they should include two adjacent middle consonants that may transpose and create another existing word, which is more frequent than the target one.

Thus, the clinical implications of the current study are: (A) look at errors and error types, and (B) use (less-frequent) migratable words in the word lists for diagnosing LPD, and, in general – include words that are sensitive to each dyslexia type in order to identify it.

## Data Availability Statement

The raw data supporting the conclusions of this manuscript will be made available by the authors, without undue reservation, to any qualified researcher. The authors maintain the rights for the reading tests.

## Ethics Statement

This study was carried out in accordance with the recommendations of Ethical Guideline of the Anadolu University Ethical Committee with written informed consent from all subjects. All subjects gave written informed consent in accordance with the Declaration of Helsinki. The protocol was approved by the Anadolu University Ethical Committee.

## Author Contributions

NF and SG conceived of the presented idea and designed the experiments. Both authors constructed the stimuli together and verified the analysis methods. NF supervised the project. SG carried out the experiments. Both authors discussed the results and wrote together the final manuscript.

## Conflict of Interest

The authors declare that the research was conducted in the absence of any commercial or financial relationships that could be construed as a potential conflict of interest.

## Footnotes

1 The age-matched control group was in fact also a reading-age matched group for many of the LPD participants: if we define a reading age control group by the total rate of errors in the non-migratable words in the screening test, the 205 age-matched control participants made an average of 1.1 errors (*SD* = 0.9), and 13 of the LPD participants performed within the normal range for their age on these words.

2 The difference between consonant-only migrations and migrations that involved a vowel letter is not due to frequency differences between the conditions, which were evenly distributed between the relative-frequency conditions. Within each frequency condition – similar frequency between target and migration counterpart, target more frequent than migration, migration result more frequent than target – the LPD participants made far more migrations that involved only consonant letters than migrations that involved vowel letters (CV and VV) 25% CC and 9% CV/VV in the frequent migration counterpart condition, 13% CC and 6% CV/VV in the similar frequency condition, and 12% CC and 3% CV/VV in the frequent target word condition.

3 We defined a target word as “clearly more frequent than its transposition counterpart” if [number of judges who judged the target as more frequent/(2^∗^number of judges who judged the response as more frequent + number of judges who judged the target and response as having similar frequency)] was larger than 1. We followed the same procedure for selecting response words as clearly more frequent.

4 A different level of familiarity, familiarity at the bigram level, did not seem to affect the participants’ errors. We calculated the bigram frequency of each of the target words in the screening test and the migratable word list that yielded a transposition response, and the bigram frequency for the transposition responses (bigram frequencies taken from Sak et al., 2008). There was no significant difference in bigram frequencies between the target words and their migration responses, *t*(452) = 0.70, *p* = 0.48.

5 This pattern differs from other types of dyslexia where morphologically complex words are more prone to errors. For example, Çapan (1989) reports on two Turkish children with dyslexia who made more errors on longer words and made many omissions in morphologically complex words. Their deficit may have been in the orthographic input buffer or the phonological output buffer, stages that are sensitive to the morphological structure of the target word (Sternberg and Friedmann, 2007; Dotan and Friedmann, 2015).
